# Switching operation and degradation of resistive random access memory composed of tungsten oxide and copper investigated using *in-situ* TEM

**DOI:** 10.1038/srep17103

**Published:** 2015-11-27

**Authors:** Masashi Arita, Akihito Takahashi, Yuuki Ohno, Akitoshi Nakane, Atsushi Tsurumaki-Fukuchi, Yasuo Takahashi

**Affiliations:** 1 Graduate School of Information Science and Technology, Hokkaido University, Kita-14, Nishi-9, Kita-ku, 060-0814 Sapporo, Japan.

## Abstract

*In-situ* transmission electron microscopy (*in-situ* TEM) was performed to investigate the switching operation of a resistive random access memory (ReRAM) made of copper, tungsten oxide and titanium nitride (Cu/WO_x_/TiN). In the first *Set* (*Forming*) operation to initialize the device, precipitation appeared inside the WO_x_ layer. It was presumed that a Cu conducting filament was formed, lowering the resistance (on-state). The *Reset* operation induced a higher resistance (the off-state). No change in the microstructure was identified in the TEM images. Only when an additional *Reset* current was applied after switching to the off-state could erasure of the filament be seen (*over-Reset*). Therefore, it was concluded that structural change relating to the resistance switch was localized in a very small area around the filament. With repeated switching operations and increasing operational current, the WO_x_/electrode interfaces became indistinct. At the same time, the resistance of the off-state gradually decreased. This is thought to be caused by Cu condensation at the interfaces because of leakage current through the area other than through the filament. This will lead to device degradation through mechanisms such as endurance failure. This is the first accelerated aging test of ReRAM achieved using *in-situ* TEM.

Resistive random access memory (ReRAM) has attracted attention for applications in next-generation non-volatile memory devices^1^,^2^,^3^,^4^,^5^,^6^. By simply applying voltage, the resistance can change between high and low resistance states (HRS and LRS). The initial state of the device is typically the HRS. The HRS converts into the LRS by applying voltage in the “*Forming*” and “*Set*” processes. Subsequent application of voltage returns the resistance to the HRS in a “*Reset*” process. Reproducible resistive switching is achieved by repeating these processes. Although voltage pulses are used in practical devices, current-voltage (*I*–*V*) measurements are also used to investigate ReRAM properties. Because of the ReRAM switch, the *I*–*V* curves are hysteretic. The resistance ratio of HRS/LRS is typically greater than 10^2^. Because of this large resistance window and *I*–*V* non-linearity, ReRAM is useful for applications in multibit and analog memories^1^,^3^,^6^ and for neural network hardware^7^,^8^.

ReRAM materials are roughly classified into three categories: perovskite-type oxides, where the electrode-oxide interface plays important role, binary oxides, where the conductive filament (CF) of oxygen vacancies contributes to the switching, and solid electrolytes with copper (Cu) or silver (Ag), which are known as conductive bridging RAMs (CBRAMs) or programmable metallization cells (PMCs)^9^,^10^,^11^,^12^,^13^,^14^,^15^,^16^,^17^,^18^,^19^. The CBRAM will be discussed in this report. The ReRAM operation of CBRAM is as follows based on electrical measurements and electronic and electrochemical considerations^1^,^5^,^12^,^13^. Assuming that a ReRAM device is composed of a Cu top electrode (TE), a solid electrolyte switching layer and an inactive bottom electrode (BE) such as titanium nitride (TiN), application of positive voltage to the TE oxidizes Cu to form cations, which move along the electric field toward the BE and are metallized there to form a CF. The CF grows toward the TE. When this CF connects two electrodes, the LRS is given (the *Set* process). The opposite reaction occurs with voltage reversal, and the CF is ruptured to achieve the HRS (the *Reset* process). However, determination of the detailed mechanism is hard to accomplish with only electrical measurements.

To understand the switching mechanism in real space, *in-situ* transmission electron microscopy (TEM) was applied^20^,^21^,^22^,^23^,^24^,^25^,^26^,^27^,^28^,^29^. Growth and rupture of a conductive Cu filament in copper-doped germanium sulfide (Cu-GeS) was dynamically confirmed with simultaneous *I*–*V* measurements in a previous study^24^,^26^. Materials with different cation mobilities have been previously studied using *in-situ* TEM and the details of the filament formation was discussed^28^. However, in the majority of these works, the sample shape was designed for TEM measurements, which is not the case in real ReRAM devices. Operation did not use *I*–*V* cycles or voltage pulses, and was slow. The current was small (less than a few μA) compared with real ReRAM devices. These experimental conditions are good enough to extract the essential switching mechanism from the complicated phenomena occurring in ReRAM switching of real devices. However, the results do not allow ReRAM device properties such as endurance^30^,^31^,^32^,^33^,^34^ and retention time to be investigated, which are important when designing ReRAM devices to be used in electronic circuits. For this purpose, multiple switching cycles with various limits of the compliance current (*I*_comp_, the limiting current in the *Set* process) should be executed on the ReRAM multilayer sample. However, such reports were quite rare^35^,^36^.

Therefore, our main purpose of this work was set to investigate the repetitive ReRAM operation and the device degradation such endurance failure using *in-situ* TEM. To achieve this investigation, the ReRAM showing clear and abrupt *Set* and *Reset* switching is required. Otherwise, the result in TEM cannot be even an analogy of real ReRAM cells showing digital switching. In previous works^11^,^12^,^17^,^18^, some oxides combined with a Cu (or Ag) electrode were reported to yield abrupt switching at low voltage (e.g. less than 1.0 V) with low current (e.g. less than 10 micro amps). These oxides can be candidates to be investigated without abrupt and eternal device destruction. Tungsten oxide (WO_x_) with a Cu electrode is one of such materials. Here, a multilayer film, where a WO_x_ layer is sandwiched between two electrodes of Cu and TiN having a stacking of Cu/WO_x_/TiN, was investigated using *in-situ* TEM to determine the degree of device degradation as well as the ReRAM operational mechanism. Ten *I*–*V* switching cycles were executed. The *I*–*V* switching properties were similar to those of a conventional device prepared using lithography. At the *Forming* process, a dark region appeared in the TEM image, which was thought to correspond to a Cu filament. In subsequent *Set*/*Reset* switching processes, no microstructural change was seen except in a process where a large negative voltage was continuously applied to the Cu electrode after *Reset* switching (*over-Reset*). With increasing *I*_comp_, ReRAM switching cycles were achieved almost without performing the *over-Reset* process. Here, a large switching current is thought to increase the local temperature and to induce leakage current in the region of the WO_x_ other than the filament. Therefore, these experiments are “accelerated aging tests” performed under severe conditions, which is usually done before practical use of electronic devices. When the switching cycles were repeated, the resistance in the HRS gradually decreased. Also, the WO_x_ layer in the TEM images became thin. This may be the mechanism of endurance degradation^30^,^31^,^32^,^33^,^34^. This is the first time that failure analysis of ReRAM was performed using *in-situ* TEM.

## Results

### Switching properties of the reference device and the TEM sample

**Figure 1:**
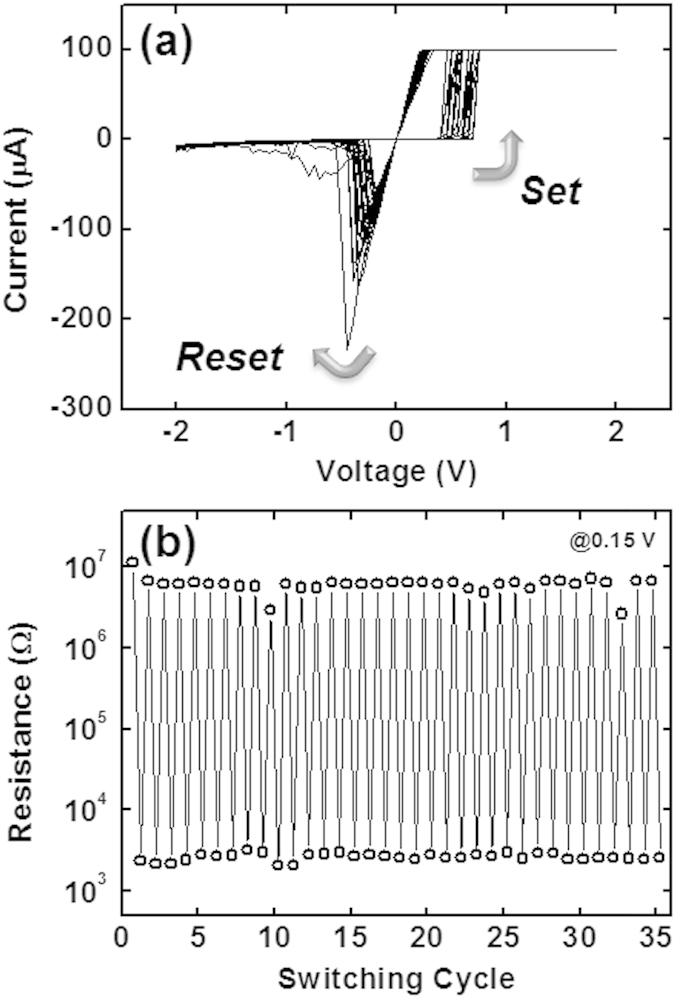
(**a**) The *I*–*V* switching cycles of a reference device (4 μm in size). (**b**) The endurance of the device evaluated at +0.15 V from (**a**). Note that the resistance of the serial resistor (1 kΩ) was added as an off-set.

First, we compared the electrical properties of a TEM sample with that of a reference ReRAM device fabricated using conventional lithography. The *I*–*V* cycles of the reference device are shown in Fig. 1(a), where switching started from the pristine state. There are 34 *Set*/*Reset* cycles shown in this figure, and typical bipolar switching was confirmed in all switching cycles. Clear and stable *I*–*V* switching curves were seen. The *Set* voltage (*V*_*Set*_) was between 0.4 and 0.7 V, and the *Forming* voltage (*V*_*Form*_) was within this range. The *Reset* voltage (*V*_*Reset*_) was between –0.2 and –0.4 V. These values are similar to those reported earlier^17^,^18^,^19^. The resistances were evaluated from this graph at +0.15 V for the forward and backward voltage sweep. The endurance properties are summarized in Fig. 1(b). The resistance window was more than 10^3^. Our Cu/WO_x_/TiN device had reasonable ReRAM properties.

**Figure 2:**
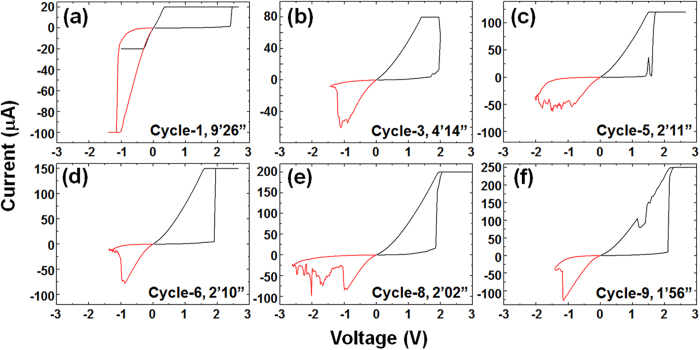
*I*–*V* curves during *in-situ* TEM when *I*_comp_ was gradually increased. The device size was about 210 nm. (**a**) Switching curve from the pristine state. (**b**)–(**f**) Switching curves in subsequent cycles. The black and red curves correspond to the *Set* and *Reset* operations, respectively. The intervals between these curves are shown in each graph.

*I*–*V* curves of the TEM sample during *in-situ* experiments are summarized in Fig. 2. Ten switching cycles were investigated while increasing the compliance current (*I*_comp_) from 20 to 300 μA. The black curves denote the *Set* process and the red curves correspond to the *Reset* process. The *Reset* process was measured at an interval after the *Set* cycle was completed. These intervals are shown in the *Set*/*Reset* graphs in Fig. 2. The red curve is smoothly connected to the black curve. Therefore, it can be concluded that the LRS retention time was longer than the interval indicated in each graph. In the *Forming* process for the first *Set* from the pristine state [Fig. 2(a)], the resistance changed to the LRS at 2.4 V (=*V*_Form_). In the subsequent (first) *Reset* process [the red curve in Fig. 2(a)], a larger current (>100 μA) than *I*_comp_ used for the *Forming* process (=20 μA) was required. This could be caused by the current overshoot after abrupt *Forming* switching. In subsequent *Set* processes, the switching voltage (*V*_Set_) was smaller than *V*_Form_. In many cases, the *Set* switching was sharp. Compared with Fig. 1(a) of a conventional device, the operational voltage was slightly high as shown in Fig. 2. This can be explained by the small device size of the TEM sample. In small devices, a number of weak points is smaller than in the large device and so conductive filaments cannot be easily formed under a low voltage^37^. The resistance of the Si substrate serially connected to the ReRAM is also a possible origin of this difference in operational voltage.

**Figure 3:**
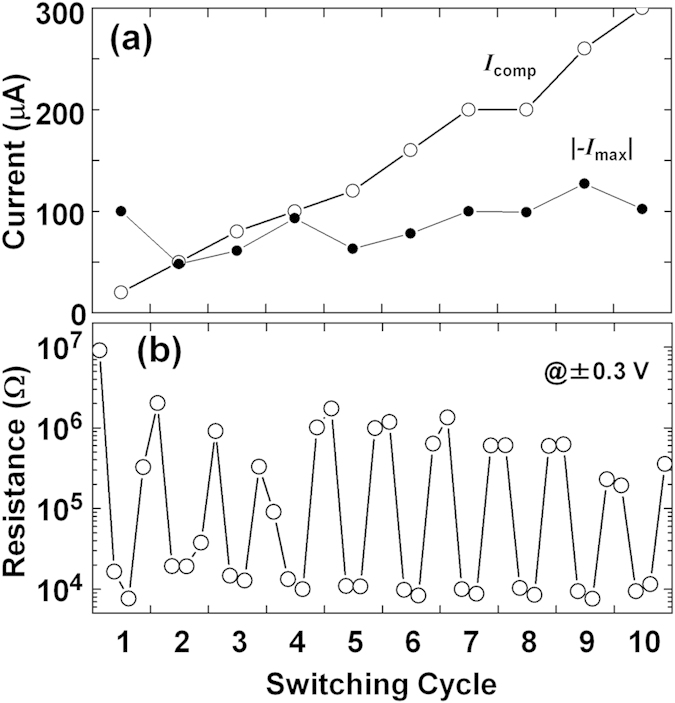
ReRAM properties during *in-situ* TEM, evaluated from the *I*–*V* curves in Fig. 2. (**a**) *I*_comp_ and |–*I*_max_|. (**b**) Endurance properties of the *Set* and *Reset* operations at ±0.3 V. In this figure, the dataset in each switching cycle contains four data points. The first two resistances were evaluated at +0.3 V in the first quadrant of the *I*–*V* graphs (the HRS and the LRS), while the last two resistances were evaluated at –0.3 V in the third quadrant (the LRS and the HRS). The minimum resistance was limited by the resistance of the substrate (~10 kΩ).

There are several reports (not using TEM) that a large *Reset* current is needed when large *I*_comp_ is used for the *Set* process^38^,^39^,^40^,^41^,^42^. This is understandable because thick filaments may be formed with a large *Set* current (large *I*_comp_) and a large *Reset* current is required to rupture thick filaments. Using the *I*–*V* graphs measured during TEM, the compliance current (*I*_comp_) and the maximum current in the *Reset* cycle (|–*I*_max_|) were determined and are summarized in Fig. 3(a). The maximum current |–*I*_max_| tended to increase with *I*_comp_. One exception was the first cycle (*Forming*/*Reset*) where the current overshoot at *Forming* induced enlargement of |–*I*_max_|. The cyclic endurance is shown in Fig. 3(b). The resistance ratio of HRS/LRS was around 10^2^, which is similarly large to those of conventional CBRAM devices.

In summary, it was confirmed that the samples investigated using *in-situ* TEM reproduced the typical properties of conventional CBRAM devices. In the next subsection, the device microstructure, especially of the filaments, during the *Set* and *Reset* switching operations investigated using *in-situ* TEM will be discussed.

### Conductive filaments in ReRAM switching cycles

**Figure 4:**
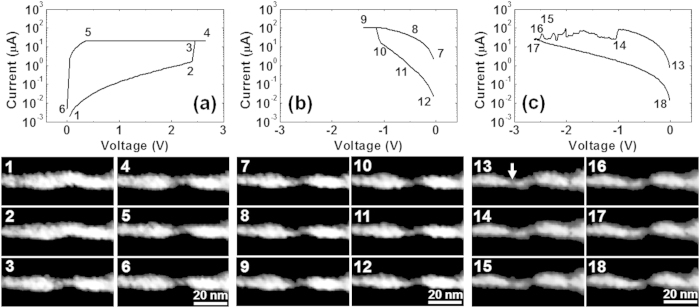
*I*–*V* curves and *in-situ* TEM images. (**a**) The first *Set* (*Forming*) cycle corresponding to Fig. 2(a). Filament evolution from the Cu and the TiN electrodes was identified (see also the video, S1.mov). (**b**) The first *Reset* corresponding to Fig. 2(a). No change was seen even though the resistance changed from the LRS to the HRS (see also the video, S2.mov). (**c**) The eighth *Reset* corresponding to Fig. 2(e). The filament with an arrow was thought to mainly contribute to electrical conduction and shrunk during *over-Reset* (see also the video, S3.mov).

*In-situ* TEM images of the first *Set* (the *Forming* process) are shown in Fig. 4(a). These images were extracted from a video (see Supplementary Information: S1.mov). While no change was seen until just before *Forming* (state 2), a dark region appeared at *Forming* (state 3). In our previous works on Cu-GeS and Cu/MoO_x_/TiN, similar image change was seen, and Cu filaments were identified using EDX analyses^24^,^26^,^35^. The conductive filament of oxygen vacancies is also expected to contribute the LRS of this ReRAM. However, it does not give dark contrast in the TEM mage. Thus, this dark region is thought to contain much Cu. While it is difficult to determine the dynamics of CF growth because the *Forming* switch occurred only within one video frame (30 ms), the CF seemed to grow from both electrodes because the contrast of both electrodes swelled into the WO_x_ layer. This cannot be explained only through an electrochemical reaction, where the CF grows from the BE to the TE^13^. Because the CF forms abruptly with an overshoot current, another factor must also contribute to CF formation, such as soft-breakdown seen in binary oxide ReRAMs^2^,^3^,^4^. Contribution of oxygen vacancies cannot be excluded because WO_x_ shows ReRAM switching even without active electrodes. As proposed by Yang *et al.*^43^, continuous generation of Cu clusters towards the TiN electrode in WO_x_, which work as bipolar electrodes, is another possibility to explain this phenomenon. After this switching, no clear change in TEM contrast was seen [states 4–6 of Fig. 4(a)] because the current was limited (20 μA). The first *Reset* operation is shown in Fig. 4(b) with a video given in the Supplementary Information [S2.mov]. While the current decreased an one order of magnitude at the applied voltage *V *= −1.1 V [between states 9 and 10 in Fig. 4(b)], there was no remarkable change in the CF image until *V*  = 0 V [states 11 and 12 of Fig. 4(b)]. Also in the subsequent *Set* process, there was no change in the image captured from the video because no CF rupturing was detected in the first *Reset*. Clear CF rupturing was seen only when the negative voltage increased after the *Reset* operation (*over-Reset*). An example of this is shown in Fig. 4(c) with the video presented in the Supplementary Information [S3.mov]. The video was recorded in the eighth *Reset* process. At *V* = −1.0 V, a weak *Reset* switching occurred [state 14 of Fig. 4(c)]. However, no clear change was seen in the image. By increasing the negative voltage to about −2.5 V [states 16 and 17 of Fig. 4(c)], the CF marked by the arrow in the image of state 13 in Fig. 4(c) started to vanish. The other CF-like region was not believed to be the path of the current, though disconnection with the electrode was not seen in the image. If this CF-like region was disconnected at the interface with the electrode as proposed earlier^13^, such disconnection can be observed using TEM only when the interface is extremely flat along the electron beam for TEM (about 200 nm in the present case). This condition was not satisfied here, and the disconnection position of the CF-like region could not be identified. In these switching series, microstructural change of the CF was only occasionally identified even with ReRAM switching. This may indicate that switching occurred only in a local region that was not detected because of interfacial roughness.

### Microstructural change with increasing compliance current

**Figure 5:**
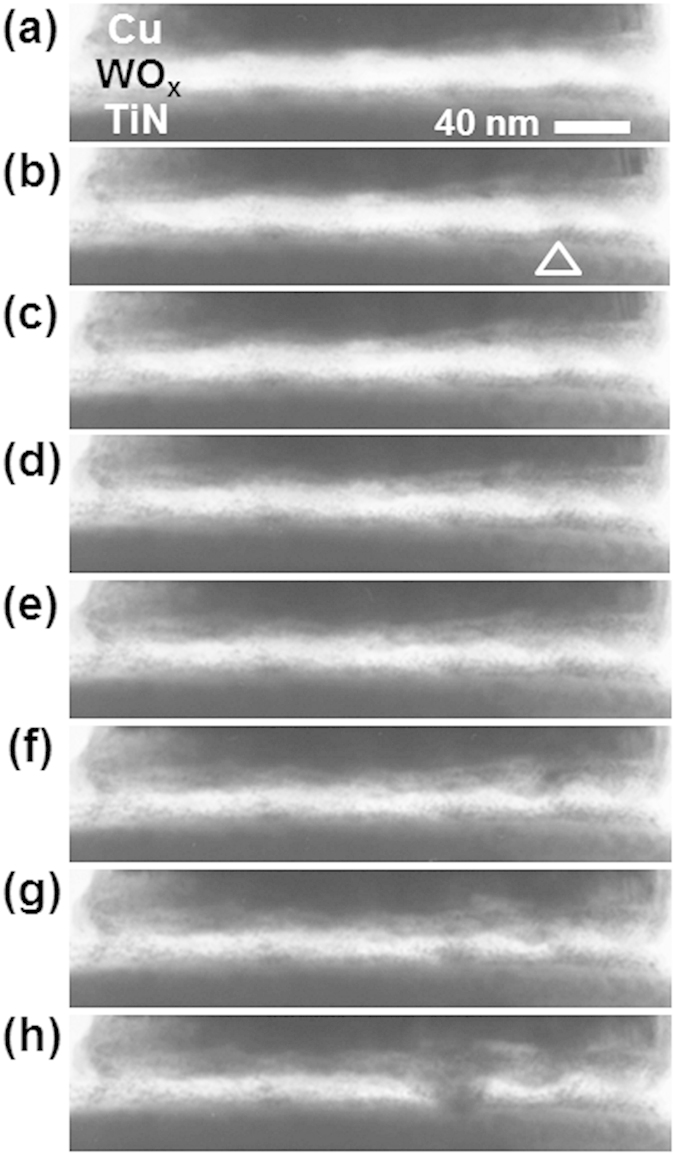
TEM images taken. (**a**) in the initial state, and after the (**b**) first (*I*_comp_ = 20 μA), (**b**) second (50 μA), (**d**) fourth (100 μA), (**f**) eighth (200 μA), (**g**) ninth (250 μA) and (**h**) tenth (300 μA) *Reset* operation. The filament thickened and its position changed. The bright region corresponding to the WO_x_ became thin. The triangle in (**b**) shows the CF position that appeared in the *Forming* process.

Looking at the cyclic endurance graph in Fig. 3(b), the resistance in the HRS gradually decreased although the resistance ratio was large. Continuing the switching cycles, the device will reach endurance failure as seen in conventional ReRAM devices^30^,^31^,^32^,^33^,^34^. Here, the microstructure in the initial state and after subsequent *Reset* operations are compared [Fig. 5].

After the *Forming* operation of the initial state [Fig. 5(a)], a CF was formed at the position marked with a triangle [Fig. 5(b)]. This corresponds to the operation shown in Fig. 4(a) (see also S1.mov). In subsequent *Reset* operations, no clear change in the CF was seen [Fig. 5(c–e)]. With further increase of the *Set* current, the growth of small deposits was identified on the TiN [Fig. 5(e–g)]. When *I*_comp_ was 300 μA, a thick CF appeared at another position [Fig. 5(f)]. This change in position was probably caused by a weak *over-Reset* process in cycle eight erasing the CF [Figs 2(e) and 4(c)]. Similar results have been reported in earlier work on Cu/MoO_x_/TiN^35^. When increasing the *Set* current by raising *I*_comp_, a thick CF appeared. This may relate to multi-level memory function. Resistance modulation could not be confirmed in this experiment because the LRS value was limited by the resistance of the Si substrate.

At the same time, by continuing the switching cycles with increasing *I*_comp_, the WO_x_ layer became thin as seen in Fig. 5. This indicates that current flowed in a wider area of WO_x_ in addition to the CF when *I*_comp_ was high. Cu moved in a wide area because of this current leakage and was deposited at the interface. This may lead to HRS endurance failure. HRS endurance failure has been reported to appear when the *Reset* process is weak relative to the *Set* process^30^,^31^. The ruptured tip of the CF was believed to be the origin of this device failure. When *I*_comp_ is large, a high resistance ratio with good retention can be achieved^36^. However, based on our result in this report, Cu tends to accumulate at the interface, not only around the CF. It was also reported that the HRS endurance failure can be recovered by operating *over-Reset* with a large current^30^. In this case also, Cu intrusion and deposition in a wide area is thought to occur. To avoid ReRAM degradation because of Cu deposition, exploring the material selection (of the switching layer and electrodes) and the device design must be important, so that the stable CF can be formed at low current.

## Discussion

The microstructure of Cu/WO_x_/TiN was investigated during *I*–*V* switching cycles using *in-situ* TEM. Here, multiple switching cycles were successfully achieved to observe the *Set* and *Reset* processes with increasing switching current. This was performed without permanent breakdown of the device at more than a hundred micro-amps. Similar studies in recent years were mainly performed with low current (less than a few micro-amps) to observe only the *Set* process. High current operation increases the local temperature around the conductive filament. Therefore such experiments correspond to the “accelerated aging tests” usually carried out in the development of electronic devices. Based on the data obtained, the process of device degradation and resistive switching were better understood.

In the *Forming* process, the Cu CF appeared to bind between the BE and the TE. The CF appeared from both the BE and TE. However, a clear change in the CF image was not identified in subsequent *Set/Reset* operations. Shrinkage and erasure of the CF was seen only in *over-Reset*. Only the local area around the CF may contribute to switching. With increasing the operational current, the filament became thick. This can be used for multi-level memorization. However, at the same time, the CF position became unstable. Also, Cu seemed to penetrate into the WO_x_ switching layer with repeated switching cycles while raising the *Set* compliance current, not only around the CF position but also in other regions. This made the switching layer thin. This may lead to reduction of the HRS resistance and further the HRS endurance failure. It is expected that the retention properties improve with increased *Set* current. The HRS/LRS resistance ratio becomes large after the *over-Reset* process because the resistance in the HRS becomes large. However, a large operational current could generate current leakage in the region other than at the CF. This leakage current may induce Cu intrusion from the Cu electrode to the WO_x_ layer as occurred prior to the *Forming* switching^43^. Based on the model proposed recently^43^, Cu ions moving along the electric field are thought to be reduced in WO_x_ to form nanoparticles (NPs). Continuing the current flow, new nucleation and growth of NPs propagates along the electric field, and the interface between Cu and WO_x_ shifts towards TiN. On the other hand, when the Cu drift rate is large with small reduction rate, Cu deposits appear at the interface of WO_x_-TiN (conventional electrochemical model^1^,^5^,^12^,^13^). Since the sputter-deposited WO_x_ films are thought to be inhomogeneous, both phenomena may occur. The leakage current generates also temperature increase due to joule heating. This can enhance the oxidation rate and the mobility of Cu while degree of this enhancement depends on the amount of the current. While an opposite reaction should occur by voltage reversal, the Cu segregation remained in WO_x_ because no strong *over-reset* was performed. Even after the filament formation, the switching layer other than the CF changes during ReRAM operations. It should not be forgotten for development of practical ReRAM memory cells. Control of the operational current is an important factor for realizing stable CBRAM cells.

## Methods

### Sample preparation and electrical measurements

**Figure 6:**
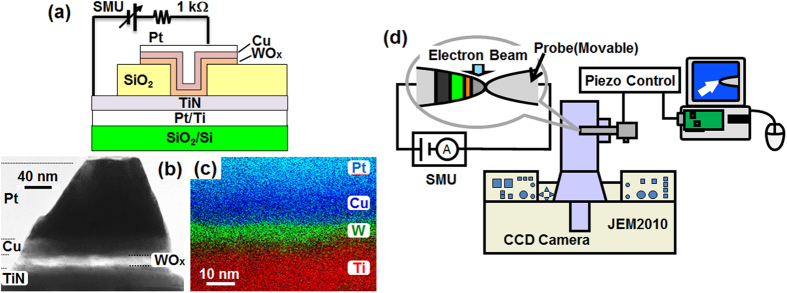
(**a**) Schematic illustration of the Cu/WO_x_/TiN ReRAM reference device. (**b**) Cross-sectional TEM image of the sample for *in-situ* TEM observations. (**c**) An example of EDX mapping made from Pt (aqua blue), Cu (blue), W (green) and Ti (red) signals. Note that the sample was different to that used in Fig. 6(a). (**d**) The experimental setup of the *in-situ* TEM.

The Pt/Cu/WO_x_ film was deposited at room temperature (RT) on TiN/Si where TiN acted as the BE. The Pt (100 nm thick) and Cu (30 nm thick) layers were deposited using RF (radio frequency) sputtering with Ar while the WO_x_ layer (20 nm thick) was prepared using reactive RF sputtering (using Ar gas with 20% O_2_). The schematics of a reference ReRAM device in the conventional device geometry is shown in Fig. 6(a). These devices were prepared by using photolithography followed by reactive ion etching (RIE) and/or lift-off. The circular contact hole was 4 μm in diameter. For *I*–*V* measurements (performed in air at room temperature using a Yokogawa GS610 source-measure-unit (SMU)), a resistor of 1 kΩ was serially connected to prevent permanent breakdown of ReRAM during *Set* switching. The TEM sample shown in Fig. 6(b) was obtained after processing using the ion shadow method^44^,^45^,^46^. Elemental mapping [from a different sample to that shown in Fig. 6(b)] is shown in Fig. 6(c), which was measured using EDX (energy dispersive X-ray spectroscopy) with a FEI Titan3 G2 STEM (scanning TEM). Clear stacking was identified.

### *In-situ* TEM experiment

Figure 6(d) shows the *in-situ* TEM system with a JEM-2010 microscope (200 kV, 10^–5^ Pa, *C*_s_ = 0.5 mm). The sample was placed in a home-made piezo holder. A movable probe was contacted to the Pt/Cu (TE), and measurements were done through Si by applying voltage to the TE with a Yokogawa GS820 SMU. The range and rate of the voltage sweep were set manually. The videos were recorded with a CCD camera (30 frames/s). The contrast of the video was enhanced non-linearly to clearly identify the faint contrast inside the WO_x_ layer. Averaging of five frames was carried out to reduce the noise. The processed videos are shown in three supplementary information files (S1.mov, S2.mov and S3.mov).

## Additional Information

**How to cite this article**: Arita, M. *et al.* Switching operation and degradation of resistive random access memory composed of tungsten oxide and copper investigated using *in-situ* TEM. *Sci. Rep.* **5**, 17103; doi: 10.1038/srep17103 (2015).

## Supplementary Material

Supplementary Movie S1

Supplementary Movie S2

Supplementary Movie S3
